# Bionic reconstruction: Restoration of extremity function with osseointegrated and mind-controlled prostheses

**DOI:** 10.1007/s00508-019-1518-1

**Published:** 2019-06-14

**Authors:** Martin Aman, Christopher Festin, Matthias E. Sporer, Clemens Gstoettner, Cosima Prahm, Konstantin D. Bergmeister, Oskar C. Aszmann

**Affiliations:** 1 CD Laboratory for the Restoration of Extremity Function, Department of Surgery, Medical University of Vienna, Vienna, Austria; 2 Division of Biomedical Research, Medical University of Vienna, Vienna, Austria; 3 Division of Plastic and Reconstructive Surgery, Medical University of Vienna, Vienna, Austria; 4 Christian Doppler Laboratory for Restoration of Extremity Function, Division of Plastic and Reconstructive Surgery, Department of Surgery, Medical University of Vienna, Spitalgasse 23, 1090 Vienna, Austria

**Keywords:** Bionic reconstruction, Interface, Prostheses, Osseointegration, Rehabilitation

## Abstract

**Background:**

Loss of an extremity at any level has a major impact on a patient’s life. Using bionic reconstruction, extremity function can be restored and the patient reintegrated into daily life. Surgical procedures including selective nerve transfer and anchoring of prostheses into bone are combined with structured rehabilitation and modern prosthetic fitting. The patient is thereby able to use the prostheses intuitively and with multiple degrees of freedom.

**Methods:**

This article presents the concept and approach for modern bionic reconstruction in detail and the relevant literature. The nerve transfer matrices for targeted muscle reinnervation (TMR) and the concept of osseointegration to optimally fit a patient with a modern prosthesis are described in detail. As a clinical example, the case of a patient who suffered from traumatic amputation and subsequently received TMR in combination with an osseointegrated implant and structured rehabilitation is presented.

**Results:**

Using bionic reconstruction, basic hand functions can be restored and bimanual dexterity can expand the range of daily activities. Besides this approach to bionic reconstruction, its advantages and disadvantages are compared to hand transplantation. The limitations and perspectives of modern bionic reconstruction are also discussed.

**Conclusions:**

Bionic reconstruction is a sophisticated method for restoring extremity function and nowadays can be considered a standard of care for all levels of upper extremity amputations. An interdisciplinary approach and structured rehabilitation are necessary to master prosthetic function to ultimately reintegrate patients into daily life.

## Amputation in the past and present

The human upper extremity and especially its most distal part, the hand, is essential for interacting with otheur environment. Due to its rich sensorimotor innervation and the resulting fine motor control, multiple degrees of freedom and dexterity, it enables tasks as simple as buttoning a shirt or using a smart phone to very complex endeavors, such as playing the piano [1]. The loss of a hand is a tragic event with severe consequences for a person’s physical and psychological well-being as well as social and work life. It impacts a person’s capability to perform activities of daily living, such as personal hygiene, environmental interaction or social interaction. Furthermore, the emotional burden associated with an amputation can lead to depression as a result of an impaired body image [2, 3]. Additionally, approximately 70% of amputees experience phantom limb pain [4]. The majority of amputations affect healthy, young male patients and result from high-energy trauma [2, 5, 6]. Considering these demographic data, upper limb loss also has severe socioeconomic implications, as many patients are unable to return to their previous occupation or to work in general [7].

Attempts to replace a missing upper limb and restore function can be dated back several centuries. Historically, it was done by fitting injured knights and soldiers with very cumbersome and heavy prosthetic devices such as iron hands. Prostheses that could perform intended movement, so-called body-powered prostheses, were first developed in the nineteenth century. The World Wars I and II of the following century led to a massive demand for upper extremity replacement to help the innumerable disabled soldiers. Consequently, considerable refinements and advancements were developed for body-powered prostheses. At the same time, the first externally powered prostheses, capable of translating residual muscle electrical activity into movement, were developed. After further improvements, these myoelectric prostheses have been clinically used since the 1960s [8]. Nowadays, refined and enhanced versions of both body-powered and myoelectric prostheses are commonly used in upper extremity reconstruction [9]. Furthermore, approximately one third of patients also use passive prostheses ranging from cosmetic hands to prosthetic tools such as hooks or devices designed for specific activities [10, 11].

In addition to prosthetic developments, advancements in microsurgery have opened the possibility for upper extremity replantation as a viable treatment option for upper extremity amputation since the 1960s. Additionally, the development of modern immunosuppressive drugs during the 1980s and 1990s in combination with sophisticated microsurgical techniques have made upper extremity transplantation a potential treatment option [12–17].

The aim of this work is to demonstrate the approach for bionic reconstruction. The nerve transfer matrices for targeted muscle reinnervation (TMR) and the concept of osseointegration to optimally fit a patient with a modern prosthesis are described in detail. As a clinical example the case of a patient who suffered a traumatic transhumeral amputation of the left arm and subsequently received TMR in combination with an osseointegrated implant and structured rehabilitation is presented. Furthermore, the article discusses the approach in view of the current literature and shows future perspectives of modern bionic prostheses.

### Clinical background part 1

The patient (male, 53 years old, married, right-handed) suffered a traumatic amputation of the left upper extremity in a work-related injury by being run over by a truck. He presented at this facility 6 months after the accident with a transhumeral amputation, moderate phantom limb pain and a neuroma in the distal stump. He was impaired in his daily life routine and dependent on the help of his wife in all activities of daily living. He consulted this department to regain independence and for reintegration into work. After discussing alternative treatment options, such as biological reconstruction, bionic reconstruction was planned.

## Myoelectric prostheses

Myoelectric prostheses have been steadily improved since their introduction in the mid-twentieth century, yet the basic principle of translating muscle electrical activity into movement has remained the same [18, 19]. A standard myoelectric prosthesis usually consists of a shaft with a socket, the connection between the device and the patient, called the man-machine interface, and a robotic hand. Typically, surface electromyography (EMG) electrodes are embedded in the socket and placed above residual muscles of the stump. These electrodes record the muscle’s electrical activity that the patient can activate voluntarily via an amplitude measurement. This is then used to control the prosthetic hand. This so-called conventional control represents the most common control scheme. To ensure intuitive prosthetic control, it is desirable to record activity from muscles that are functionally related to the associated movements performed by the prosthesis. This may be done by using the antagonistic flexor and extensor muscles of the stump to facilitate closing and opening of the prosthetic hand [20, 21]. If several residual muscles are available, it opens the possibility of controlling multiple functionally related degrees of freedoms [22]. If the number of recordable muscle signals does not meet the requirements to simultaneously control multiple degrees of freedom, the use of sequential or multistate controllers to cycle between different functions by co-contracting a muscle pair poses a viable, yet unintuitive and cumbersome alternative [21, 23].

## Man-machine interface

The basis for correct functioning and therefore a major influence regarding patient satisfaction and acceptance is a stable, reliable man-machine interface, capable of translating neural information of intended movement into prosthetic control signals [24]. From an engineering point of view, the interface comprises all the elements between a machine and a human necessary to translate biological activity into electrical control signals, such as electrodes, wires and processors [25]. Due to its easy application and noninvasiveness, surface EMG is the most common approach used for prosthetic control; however, the recorded signals are greatly influenced by different electromechanical factors such as electrode displacement as well as lack of reproducible placement after donning and doffing, skin conductivity due to sweating, amount of underlying adipose tissue and movement artifacts. Furthermore, technical limitations such as amplitude cancellation or muscular cross-talk also negatively impact the recorded signal’s information. Lastly, surface EMG does not accurately represent the activity of individual motor units and therefore neural information transmitted by motor neurons but rather muscular activity [26–28]. Many of these issues may be overcome by using high-density multichannel surface EMG electrodes, implantable EMG electrodes or a combination of both [29]. Implantation of EMG electrodes reduces or eliminates the influence of the aforementioned electromechanical factors, muscular cross-talk and enables more selective access to the neuromuscular information of deeper parts of the muscle at the cost of increased invasiveness [23]. The use of high-density EMG arrays with both surface and implantable electrodes in combination with decomposition algorithms has been proposed as a method to accurately record the activity of individual motor units and has shown promising results [22, 26, 30].

Myoelectric control systems are based on the fact that muscles essentially serve as biological amplifiers for the neural information of movement transmitted via motor neurons. Instead of relying on this amplifier, several attempts have been made to interface peripheral nerves to directly extract this information. This is accomplished by implanting nerve electrodes and using the recorded neural activity for prosthetic control. Many nerve electrodes with varying degrees of selectivity and invasiveness for interfacing the peripheral nervous system have been proposed in the literature [25, 31]. Although it has been demonstrated as a potential alternative to EMG-based control, this approach remains experimental and issues regarding the amount of controllable degrees of freedom, potential nerve damage and signal-to-noise ratio are yet to be solved [31–34]. While the clinical relevance of direct neural prosthetic control for upper extremity reconstruction remains unclear, providing sensory feedback via direct peripheral nerve stimulation has been investigated for decades [35, 36]. It has repeatedly been shown that this approach is capable of eliciting sensory feedback [34, 36–39]. Despite it being mainly confined to experimental laboratory settings as of now, direct nerve stimulation could potentially become a viable approach for providing sensory feedback [40].

## Biological reconstruction—an alternative?

The development of vascular anastomosis techniques, the introduction of the operating room microscope as well as advancements and refinements of surgical tools and materials in the middle of the last century, laid the groundwork for upper extremity replantation and subsequent transplantation. The introduction of immunosuppressive drugs paved the way for hand transplantation with the first report in 1964 but with subsequent reamputation. The first generation immunosuppressants did not prevent acute rejection, therefore, it was not until the late 1990s and the advent of modern immunosuppressive drugs, that the first successful hand transplantation was performed. Despite more upper extremity transplantations (including below and above the elbow) performed in the following years, it has remained a fairly rare procedure with only 107 reported transplantations worldwide [14], with a reamputation rate of 17% [41]; however, it is absolutely crucial to carefully evaluate the risks associated with life-long immunosuppression. Furthermore, long-term success is highly dependent on the patient’s psychological status and compliance, as the presence of a donor hand may be an unbearable psychological burden and failing to adhere to the immunosuppressive regimen may result in transplant rejection [14, 15]. The level of amputation is another important factor to consider. With nerves regenerating at about 1 mm/day and a maximum time frame of 18 months for reinnervation before irreversible distal muscle fibrosis, above elbow transplantations often result in impaired hand function and even functionless hands due to the long regeneration distance. It has been argued that prosthetic fitting and not hand transplantation should be the standard treatment especially in cases of unilateral below elbow amputation due to fewer risks as well as the ability to compensate for functional deficits [42]. Despite the many potential benefits of upper extremity transplantation, it is also important to consider the financial burden associated with the procedure in the final treatment decision. The costs of a transplantation (especially life-long immunosuppression) are significantly higher than with a prosthetic fitting. Some studies suggest that, from an economical perspective, prosthetic fitting may be the preferred strategy depending on the type and level of amputation [43, 44].

### Clinical background part 2

After detailed examination of the patient and the stump, surface EMG testing to check for potential muscle signals, as well as a psychological assessment were conducted. Together with the patient it was then decided to perform targeted muscle reinnervation in order to create an intuitive functional interface. The surgery was planned according to the nerve transfer matrix with the overall goal to increase the number and quality of myoelectric signals for prosthesis control.

## Targeted muscle reinnervation (TMR)

In addition to technical refinements, surgical procedures such as TMR are also capable of increasing the number of available EMG signals [45–47]. This procedure is based on selectively transferring remaining peripheral nerves to remnant stump muscles. For example, the median, ulnar, radial and musculocutaneous nerves can be transferred to different anatomical parts of the pectoralis major muscle. Following sufficient recovery and rehabilitation, the patient is then able to volitionally elicit contractions of specific parts of the reinnervated muscle by attempting to open and close the hand or flex and extend the elbow. The muscular activity is then recorded and used for myoelectric prosthetic control potentially enabling simultaneous control of two or more degrees of freedom [48]. The current standard operating matrix for TMR nerve transfers is demonstrated in Table 1.

**Table 1:** Nerve transfer matrix for targeted muscle reinnervation at a transhumeral level (modified from [52])

Nerve	Target muscle	Prosthetic function
Musculocutaneus nerve	Caput longum m. biceps brachii	Elbow flexion
Ulnar nerve	Caput breve m. biceps brachii	Hand closing
Median nerve	M. brachialis	Wrist pronation
Radial nerve	Caput longum/mediale m. triceps brachii	Elbow extension
Deep branch of the radial nerve	Caput laterale m. triceps brachii	Hand opening
Deep branch of the radial nerve	M. brachioradialis	Wrist supination

**Fig. 1:**
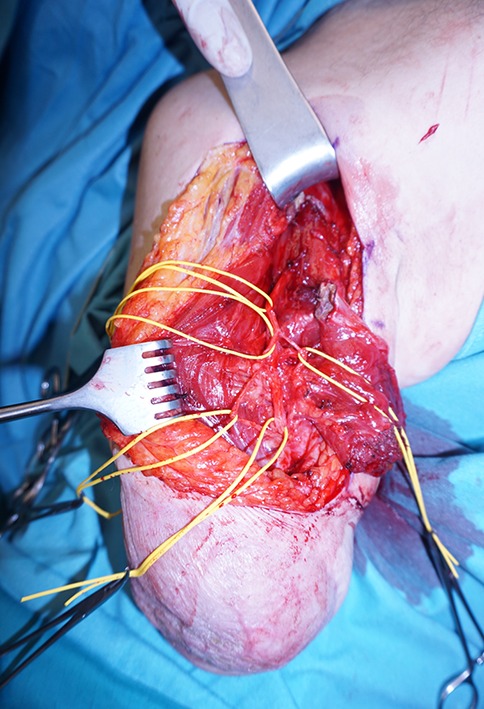
Intraoperative situs of targeted muscle reinnervation. Marked are the musculocutaneus nerve and its branches to the long and short head of the biceps and brachial muscle as well as the N. cutaneus antebrachii lateralis

Furthermore, sensory nerve fibers of the transferred brachial plexus nerves reinnervate the overlying skin mapping the missing limb’s sensitivity onto it. This phenomenon is called targeted sensory reinnervation and opens the possibility of providing natural, intuitive sensory feedback to the patient [49]. These selective nerve transfers have implications both for the peripheral and central nervous system. First, patients who underwent TMR and sensory reinnervation surgery show close to normal cortical representation of the missing limb several years later which may play a role in preventing or diminishing phantom limb pain and neuroma formation [50]. Secondly, the nerves transferred during TMR typically contain more motor neurons than the original ones. This leads to hyperreinnervation of the targeted muscle, more and smaller motor units and therefore potentially more separable myoelectric control signals ([29, 51]; Fig. 1).

## A perspective on regaining sensation

As previously discussed, a lot of effort has been directed towards improving myoelectric prosthetic control and therefore functionality. Although research has shown impressive results in this respect, one major drawback of myoelectric prostheses compared to transplantation is the lack of natural sensory feedback, which remains one of many important demands of prosthesis users and contributes to user dissatisfaction as well as prosthesis abandonment [9, 53]. Sensory feedback has a great impact on a patient’s sense of embodiment, reduces phantom limb pain and potentially improves control [40]. Various approaches to providing sensation have been described in the literature. Noninvasive methods include sensory substitution, such as vibrotactile or electrotactile stimulation and modality-matched feedback applied to the patient’s skin [54]. An example of the latter is the transmission of pressure measured at the prosthetic hand to the patient’s skin applied via a pneumatic mechanism or small motors [55, 56]. Although research suggests that these approaches may potentially increase overall functionality, they remain relatively unintuitive and unnatural [54]; however, the combination of noninvasive methods with targeted sensory reinnervation may overcome these limitations and pose a viable option for sensory feedback [49].

The idea of direct peripheral nerve stimulation to elicit more natural and intuitive sensory feedback was first attempted several decades ago and has been in the focus of current research to restore sensation [36, 57]. Various types of neural interfaces have been experimentally implanted into patients and used to stimulate peripheral nerves eliciting different sensations, such as pressure, tapping, vibration, tingling, paresthesia, pain as well as proprioceptive feedback [34, 37–39, 58]; however, these approaches are largely experimental, have been performed in very few patients and did not investigate long-term safety and durability [59].

**Fig. 2:**
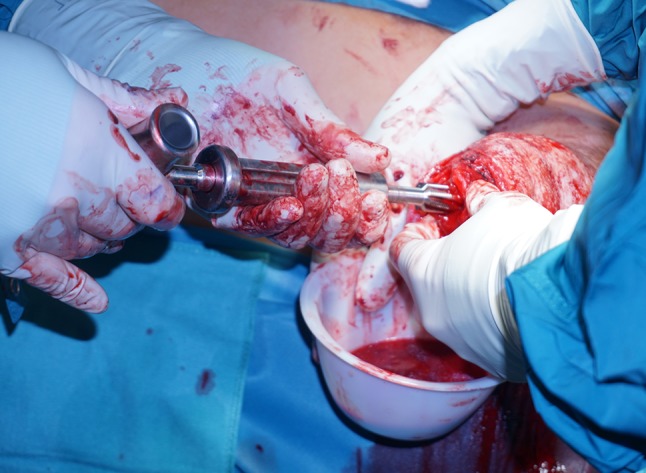
Bone preparation for implant insertion by manually drilling longitudinally into the remaining part of the humerus

Currently, commercially available myoelectric prostheses still do not offer any form of intentional sensory feedback ([54]; Fig. 2).

### Clinical background part 3

**Fig. 3:**
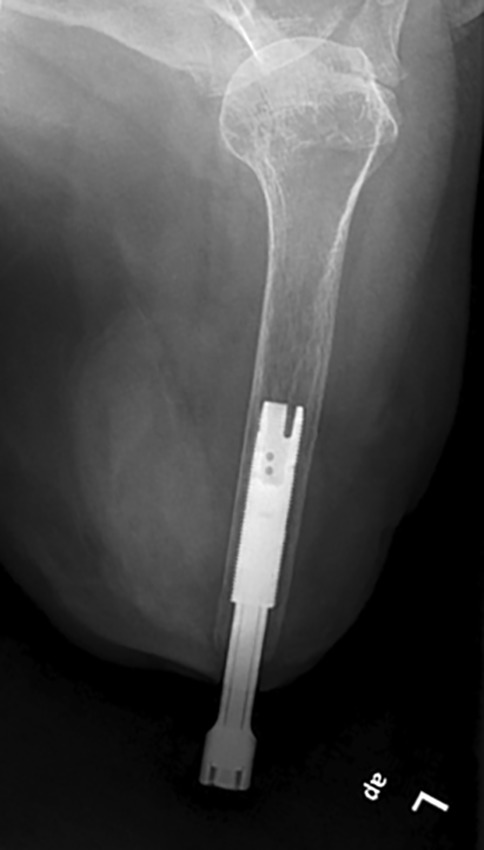
Postoperative control X‑ray of the osseointegrated implant in the humerus

**Fig. 4:**
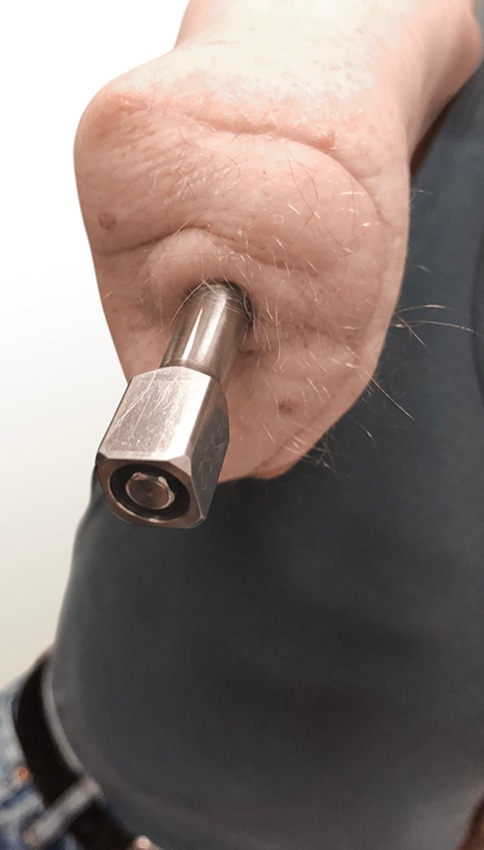
Amputation stump with the osseointegrated implant to connect the prosthesis directly to the skeleton

In addition to TMR, osseointegration was chosen to directly anchor the prosthesis in the human skeleton. A preoperative X‑ray as well as computed tomography (CT) scan were performed to evaluate bone tissue as well as implant size. The TMR was performed during the same operation in which the implant was placed into the bone (Figs. 3 and 4).

## An alternative to the conventional stump-socket interface

Ideally, the socket should establish a stable mechanical connection between the user and the prosthesis to ensure reliable control signal acquisition, unrestricted range of motion, even pressure distribution and therefore comfort and functionality. Many unresolved issues, such as excessive heat and sweating, unequal pressure distribution, skin irritation, ulceration or even chronic infection still exist, resulting in low user satisfaction and permanent prostheses rejection [9, 24, 60, 61]. Especially short stumps are prone to some of these issues due to prostheses requiring additional fixation restricting movement [40] and potentially causing even more heat and sweating. Several of these issues may be overcome by a permanently anchoring the prosthesis into the patient’s bone in an approach called osseointegration. Originally used in dental and craniofacial surgery, osseointegration was first introduced as an option for extremity reconstruction in 1990. Starting with the treatment of a bilateral, transfemoral amputee, this novel reconstructive approach was then used to treat several other transfemoral as well as thumb and transradial amputees. After improving the surgical techniques and standardizing the entire procedure including rehabilitation in the following years, osseointegration now represents a viable option for treating various types and levels of amputations. This is done by using two titanium implants, namely a fixture and an abutment. First, the fixture is implanted into the intramedullary cavity of the stump bone (for example the humerus or femur). The abutment then percutaneously extends from the fixture and serves as an anchor for the prosthesis. Compared to conventional stump-socket interfaces, osseointegration establishes a stable mechanical connection, prevents common skin-related problems such as sweating, irritation and ulceration while maintaining full range of motion. Furthermore, it provides a certain degree of sensory feedback through a phenomenon called osseoperception in which tactile sensations are transmitted to the bone via the anchorage and can be perceived by the patient [62, 63].

Thumb, transradial, transhumeral as well as transtibial and transfemoral implants have been employed with impressive recovery, functionality and long-term success [64]. Despite overcoming most issues related to conventional stump-socket interfaces, the transcutaneous abutment bears the risk of soft tissue infections and other stoma-related complications. Furthermore, mechanical complications such as implant loosening, device breakage or bone fractures have been reported [65].

In addition to the aforementioned benefits, osseointegration may also serve as a bidirectional communication interface between the patient and the prosthesis. In a recent study, leads from electrodes implanted into the residual muscle and nerve tissue of the stump were led through the titanium implants and connected to the prosthesis enabling both stable and reliable long-term prosthetic control as well as sensory feedback [39].

### Clinical background part 4

**Fig. 5:**
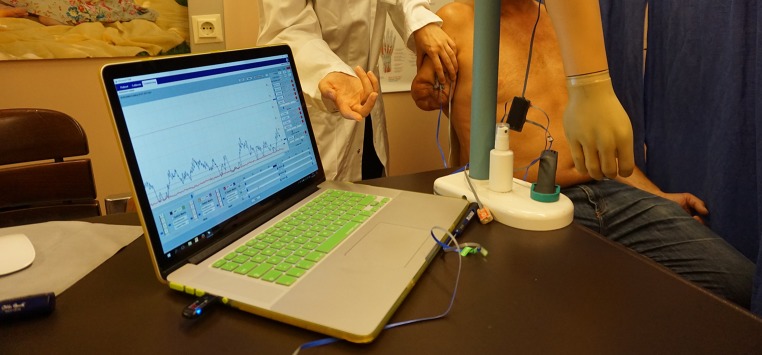
Surface EMG signal training with visualization of the signals for optimal rehabilitation

Following the implantation and TMR, a structured rehabilitation protocol was applied for TMR signal training as well as loading of the osseointegrated implant. Weight loading of the implant was carefully started 1 month after surgery and 2 months after surgery, the patient was already able to elicit two distinct myosignals. Details of the structured rehabilitation protocols have been published previously [64, 66]. After 5 months postoperatively the patient is already able to load maximum training weight onto the implant and to consciously activate four muscle signals for prosthetic control. A prosthesis was fitted and is now used in daily activities (Fig. 5).

## Rehabilitation

A structured training and rehabilitation program is essential to master the full potential of the prostheses and its various control schemes. Patients should undergo rehabilitation before and after prosthetic fitting to establish adequate muscular control signals, as has been described in the bionic reconstruction algorithm [67]. Furthermore, physical therapy should include care of the residual limb to maintain the full range of motion of its joints. It is important to note that prosthesis acceptance and long-term use are higher among patients who received specialized prosthetic training [68].

The process of learning to volitionally produce separate, stable, repeatable muscular activity before prosthetic fitting, especially after nerve transfer surgery, can be supported by training and rehabilitation methods such as EMG biofeedback with visualization of the elicited signals [69, 70]. This is followed by fitting the patient with a diagnostic socket and prosthesis to train the different EMG signals with the corresponding prosthetic functions. Subsequently, the prosthesis should also be incorporated into activities of daily life. After sufficient training, the patient is fitted with the final socket and prosthesis for long-term use; however, it is important to regularly follow up the patient’s prosthetic function as well as quality of life and adjust the socket if needed [71, 72].

**Fig. 6:**
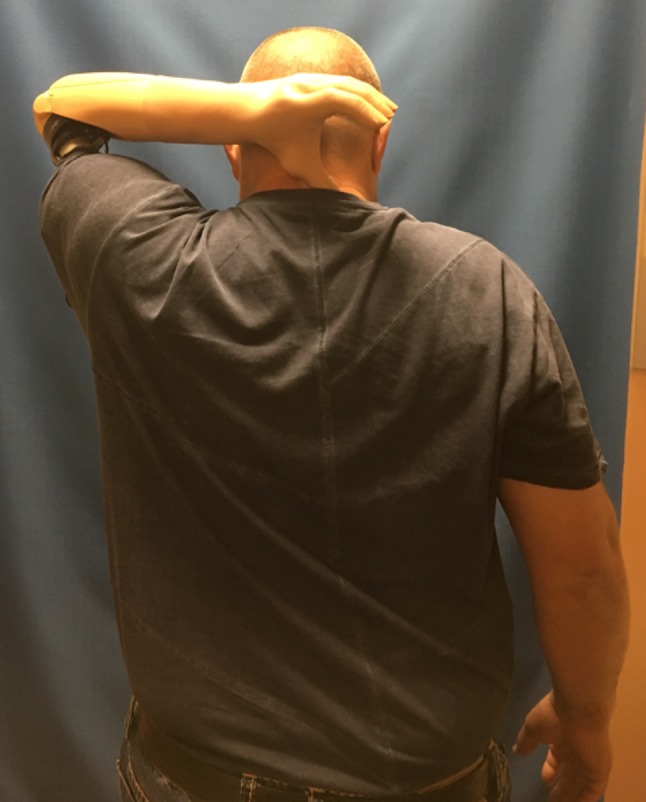
After bionic reconstruction, the patient gains independence in daily life activities. Due to osseointegration, an impressive range of motion is accomplished in the shoulder joint. With a standard prosthesis with traditional shafts, this range of motion cannot be achieved

The use of video games or virtual reality has also been proposed for rehabilitation purposes and recent evidence suggests that these approaches enable easy, motivating and correct training and may improve user acceptance as well as satisfaction and therefore decrease prostheses abandonment ([73–76]; Fig. 6).

## Conclusion and future perspectives

Bionic reconstruction poses a promising, cost-efficient treatment option for the restoration of upper extremity function. Compared to upper extremity transplantation, it comes without the risks of life-long immunosuppression and similar functional outcomes under certain circumstances but still lacks natural sensibility [42]. Therefore, bilateral amputation is still an indication for transplantation; however, establishing natural and intuitive control of myoelectric prostheses as well as sensory feedback will likely require more research, development and refinement. This is mainly due to limitations of current man-machine interfaces. Most commercially available prostheses operate with conventional surface EMG control which is unable to accurately extract the neural information of intended movement and exhibits many issues regarding signal recording stability and reliability [26–28]. Technical advancements such as high-density electrode arrays and implantable electrodes, advanced pattern recognition algorithms as well as TMR have shown impressive functional results and may become viable clinical solutions [40]. With respect to sensory feedback, direct peripheral nerve stimulation bears the potential to become a solution due to it eliciting sensations while also improving phantom limb pain and increasing a patient’s sense of embodiment; however, issues regarding long-term stability and safety have to be addressed first [40, 59].

In addition to efforts directed at improving both muscle and nerve interfacing, more and more attention has been paid to the challenges surrounding the conventional stump-socket connection. Issues such as skin irritation, discomfort and restricted range of motion may be overcome with bone-anchored prostheses. Osseointegration has been used to treat various types of amputations. Furthermore, it opens the possibility of a stable and reliable bidirectional communication interface by leading the wires of implanted muscle and nerve electrodes through the bone anchor components to the myoelectric prosthesis [39].

To make full use of all the mentioned surgical and technical approaches, it is essential to provide the patient with structured training and rehabilitation programs as described in the bionic reconstruction algorithm [67]. Innovative training methods such as augmented reality or video games may help in improving the patient’s motivation and subsequently increase prosthesis acceptance [73, 74, 76]. Ideally, experts from the different fields necessary to provide all the aforementioned possibilities for optimal prosthetic care should be united in centers specialized in extremity reconstruction. This would enable highly efficient translational research and subsequently provide patients with optimal treatment to restore extremity function.
